# Energy transfer and influencing factors in soil during compaction

**DOI:** 10.1371/journal.pone.0242622

**Published:** 2020-11-20

**Authors:** Jie Li, Xiaohong Bai, Fuli Ma

**Affiliations:** 1 College of Civil Engineering, Taiyuan University of Technology, Taiyuan, Shanxi, China; 2 Shanxi Key Laboratory of Geotechnical and Underground Engineering, Taiyuan, Shanxi, China; Soil and Water Resources Institute ELGO-DIMITRA, GREECE

## Abstract

In China, large-area excavation and filling engineering has increased rapidly with the expansion of construction land. The quality of filling engineering is the most important guarantee for the stability of building structures. Among all research on fill soil, the compaction characteristics are significant for indicating the strength and stability of filling engineering. In this paper, two layers of loess fill soil were compacted by a self-manufactured test system with three different compaction energies. Based on the variation in the soil bottom pressure obtained in the tests, the influence of the compaction parameters on the soil bottom pressure was investigated. The results show that the compaction curve can be used instead of the curve of the change in soil bottom pressure with water content; as the soil density increases, the soil bottom pressure increases to the maximum. The relation of the energy consumption ratio of the soil bottom (***σ/σ***_***z***_) and the number of soil layers is exponential and reveals the stability of the soil skeleton formed during compaction. This paper describes the compaction characteristics of loess fill soil from the perspective of energy transfer, and the conclusions provide a theoretical basis for soil filling engineering.

## Introduction

Currently, there is a large amount of filling engineering for the expansion of construction land in China [1]. Many serious problems hinder the process of controlling soil strength and stability during compaction. For example, various engineering distresses, such as pavement cracking due to insufficient fill strength, have emerged one after another in Handan, China [2]. In the Yan'an and Lanzhou areas in China, the fill thickness of some projects is >50 m, and some projects have already shown deformation and destruction [3]. Due to the deficient compaction of roadbeds in the Beijing-Kowloon railway, many roadbed subsidence events and other distresses have occurred [4]. Therefore, to reduce the engineering distresses of fill, compaction parameters must be strictly controlled. However, accurately determining the degree of compaction is a crucial subject in filling engineering. The compactness is often used to represent the quality of the fill. Compactness is the ratio between the field compaction density and the maximum laboratory-tested dry density [5]. The compaction method utilized, such as roller compaction, tamping, and vibrating compaction, also affects the compaction quality. Regardless of the method, the soil is compressed to the required density by compressing the soil pores. The maximum laboratory-tested dry density is the peak value of the compaction curve (that is, the relationship between water content and dry density) obtained from the heavy compaction test in the laboratory. For the sake of calculating the compactness and accurately controlling the compaction parameters, many researchers have investigated the compaction process and mechanism of fine-grained soil. On the basis of summarizing multiple engineering practices, Proctor [6] emphasized the lubricating effect of water in soil during compaction. Hogentogler [7] discussed the compaction mechanism from the perspective of the viscosity of water in soil. Lambe [8] explained the compaction mechanism combined with the physical and chemical effects of the soil structure and particle surfaces. Olson [9] summarized the above theoretical basis and proposed the theory of effective stress to explain the compaction mechanism.

Many factors affect compaction quality. Gurtug and Sridharan [10] proposed the use of the plasticity limit based on soil to determine the optimal water contents and maximum dry densities of soil samples under different compaction energies. Noor et al. [11] proposed a similar method using fine-grained soil as the research object. Yang [12] studied the change in compacted loess under different compaction energies and water contents and reported that the compaction quality depended on strict control of the water content. It is particularly important to determine the optimal water contents and the maximum dry densities of soil samples. Mitchell [13] studied loess under a vibrating load and reported that the number of vibration compactions significantly affected the shear strength of loess. Liu [14] performed compaction tests with six types of compaction energy and proposed the existence of “economic compaction work.” Jiang [15] analyzed loess fill after compaction through laboratory geotechnical testing and proposed that the dry density, water content, and other factors influenced the strength and deformation of compacted loess. Many experts have analyzed the mechanical properties of compacted soil. Xiao et al. [16] studied the mechanical properties of silt soil, showing that the soil samples were not well graded with a large air volume after compaction, which impeded compaction. Jia et al. [17] found that the cohesive force of compacted loess increased with increasing compactness. Lu et al. [18] studied high-liquid-limit clay using numerical simulation to analyze the influence of the initial compactness of fill soil on the stability of embankment slopes.

Extensive research has been done on the factors that affect the compaction effect and the mechanical properties of fill soil after compaction. However, the current knowledge in energy transfer in soil during compaction remains insufficient, such as: how does compaction energy pass through the soil and how do compaction parameters affect energy transfer. In this study, compaction tests were performed under different compaction energies and soil layers to test the soil bottom pressure during compaction. From the perspective of energy transfer, this paper studies the relationship between compaction impact force and soil bottom pressure under different compaction energies and soil layers and determines the energy transfer process under various compaction conditions.

## Materials and methods

The soil samples were obtained from a loess fill site in Ningwu County, Xinzhou City, Shanxi Province, China, with the geographical coordinates of 111°50'-120°40' east longitude and 38°31'-39°8' north latitude. Shanxi Province, China, and dried naturally. The basic physical properties of the soil samples, measured in accordance with “standard for soil test methods” [19], are listed in Table 1. The specific gravity of the soil particles (*d*_*s*_) was 2.70. The liquid limit (*W*_*L*_) and the plastic limit (*W*_*P*_) was 25.5% and 16.6%, respectively. The plasticity index (*I*_*p*_) of the soil was 8.9 ≤ 10, and its particle content with particle size greater than 0.075 mm was 1.8% < 50%, so it is named silt according to the “Code for Design of Building Foundation” [20].

**Table 1:** The basic physical properties of the tested soil.

Grain size (mm)/%				Specific gravity *d*_*s*_	Liquid limit *W*_*L*_/%	Plastic limit *W*_*P*_/%	Plasticity index *I*_*P*_
0.25~0.075	0.075~0.05	0.05~0.005	<0.005				
1.8	37.4	52.6	8.2	2.70	25.5	16.6	8.9

Compaction testing was conducted using a JDS-3 standard portable compactor. JDS-3 standard portable compactor is used to determine the relationship between soil density and water content, thereby determining the maximum dry density and the corresponding optimal water content. The hammer weight can be changed from 2.5kg to 4.5kg. The weight and fall height can be adjusted to suit different test requirements. The diameter of the cylinder is 152 mm, which is three times of the diameter of the hammer, allowing the lateral diffusion of energy to a certain extent. In the test, the compaction energy ***E*** (kJ/m^3^) and the number of soil layers ***n*** were given. According to the volume ***V*** (m^3^) of the solid cylinder, the hammer gravity ***W*** (kN×10^−3^), and the drop distance ***d*** (m) of the hammer, the number of hits ***N*** required for each layer of soil can be calculated based on formula (1), with the required compaction parameters listed in Table 2.

$$N=\frac{\mathrm{EV}}{\mathrm{Wdn}}$$ (1)

**Table 2:** The compaction parameters under different compaction energies.

Compaction energy *E* (kJ/m^3^)	Hammer gravity *W* (kN×10^−3^)	Drop distance *d* (m)	Solid cylinder volume *V* (m^3^)	Soil layers *n*	Number of hits *N*
1208.2	45	0.457	2.1×10^−3^	3	42
				5	25
2013.7	45	0.457	2.1×10^−3^	3	70
				5	42
2684.9	45	0.457	2.1×10^−3^	3	94
				5	56

To study the effects of different compaction energies and soil thicknesses on the energy transfer, three compaction energies (***E***) were selected: 2684.9 kJ/m^3^, 2013.7 kJ/m^3^, and 1208.2 kJ/m^3^. Each of compaction energy was applied to soil samples with two different configurations of three layers and five layers, yielding six groups of compaction tests.

The soil samples were prepared by the dry method; the air-dried soil sample was mixed evenly and sifted to obtain particles with a maximum size of 2 mm. Based on the plastic limit of the soil sample, the water content was estimated to be within the preparation range. Four groups of different water contents were prepared in each test group. According to the quality ***m*** (g) of the dry soil sample, the water content ***w***_***0***_ (%) of the dry soil sample, and the estimated water content ***w*** (%) of the four groups of soil samples, the amount of water ***m***_***w***_ (g) needed for each group of soil samples was calculated by formula (2). During preparation, the dry soil and water were fully stirred and mixed before being placed in a moisturizing dish for 24 h to ensure that the water was evenly distributed in the soil.

$$m_{w}=\frac{m}{1+0.01w_{0}}\times 0.01(w-w_{0})$$ (2)

The prepared soil samples of each group were tested via the six described testing groups according to the compaction parameters given in Table 2.

**Fig 1:**
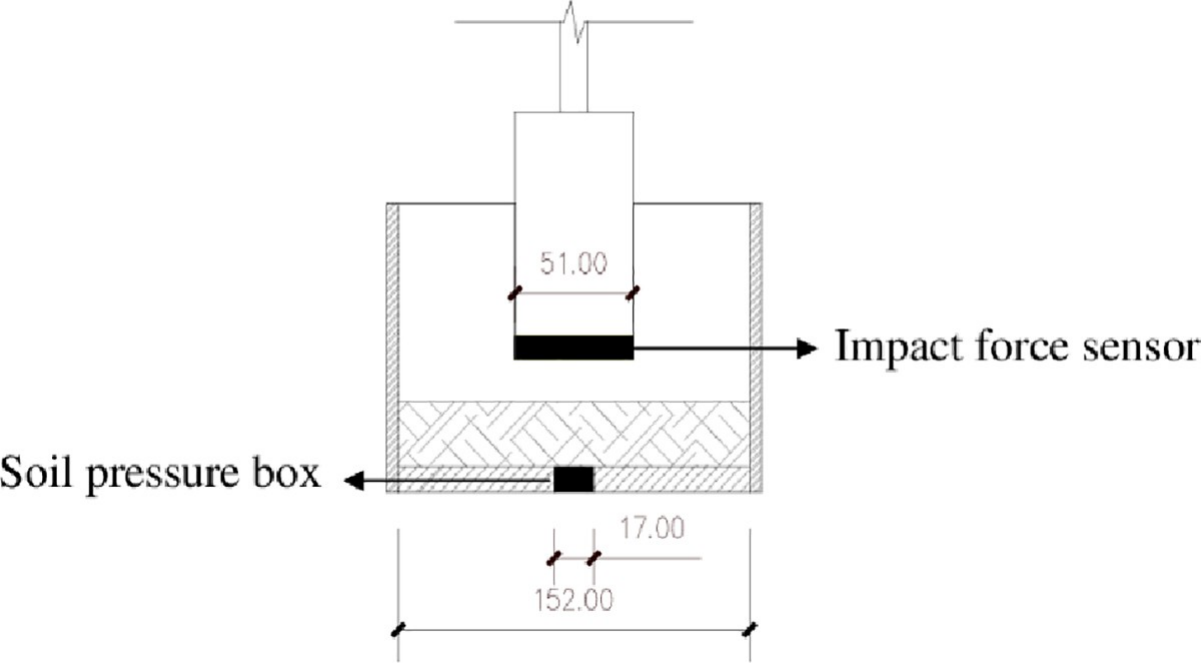
System connection diagram.

To analyze the energy transfer in the soil during compaction, this process was improved relative to that of traditional portable compactors. A soil pressure box (DZ-I) was laid at the bottom center of the compaction instrument to measure the pressure transferred to the bottom of the soil during hammer compaction. The impact force sensor (KC8731), which natural frequency is greater than or equal to 40 kHz and charge sensitivity is 3.6 PC/N, was placed at the bottom of the hammer to measure the impact force when the hammer compacted the soil. The test system is shown in Fig 1, with the mark length unit of millimeters. The black part is the location of the soil pressure box and the impact force sensor. The sensor is connected with the strain gauge (KC8951) and the data acquisition instrument (KC7703-12) for testing. The strain gauge sensitivity is 0.5V/100με and frequency response is DC~100kHz, the data acquisition instrument maximum sampling frequency is 1k~500k sample per second. The overall sampling reliability is higher.

The compaction procedure is the same as that of the compaction test in the laboratory. To eliminate the boundary effect, this study only reports the pressure of the soil bottom and the impact force of the compaction hammer of the compaction cylinder center under different compaction conditions. The current standard of most countries at home and abroad [20–22] adopts laboratory compaction test similar to this paper to obtain the maximum laboratory dry density index, so as to compare with the field dry density. Therefore, it is necessary to study how energy is transferred during laboratory compaction. According to the standard for soil test methods [19], the last stroke of each cycle hits the middle of the soil sample during compacting; 14 strokes are defined as one cycle. Therefore, to accurately measure the soil bottom pressure at the center, the measurements under different conditions are listed in Table 3.

**Table 3:** The number of stress tests *n*_*1*_ in each layer.

Compaction energy *E* (kJ/m^3^)	Soil layers *n*	Number of stress tests *n*_*1*_
1208.2	3	3
1208.2	5	1
2013.7	3	5
2013.7	5	3
2684.9	3	6
2684.9	5	4

## Results and discussion

**Fig 2:**
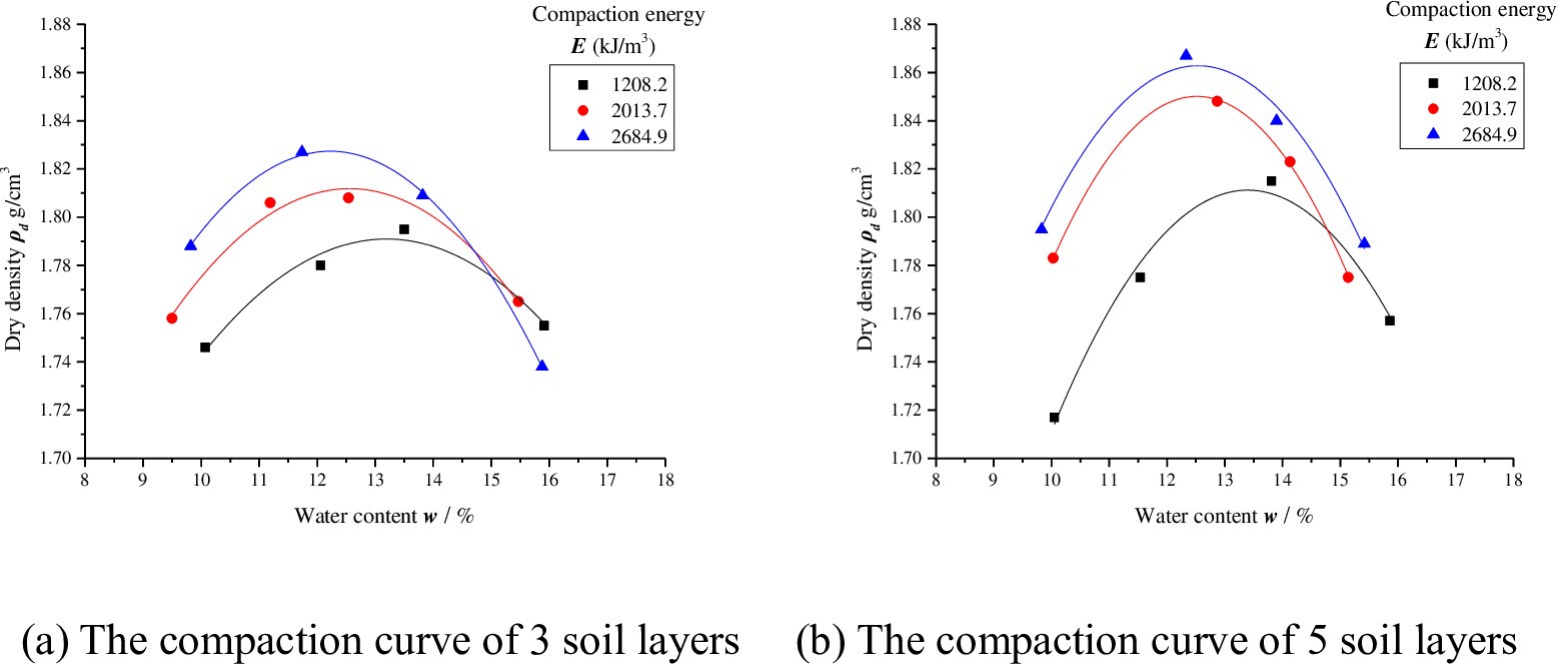
Two types of soil layers compaction curve under different compaction energies (*E* = 1208.2 kJ/m^3^, 2013.7 kJ/m^3^, 2684.9 kJ/m^3^).

The compaction test results of the six groups are listed in Table 4, and the compaction curves are shown in Fig 2(A) and 2(B).

**Table 4:** The compaction test results under different compaction energies and layers.

Soil layers *n* *E* (kJ/m^3^) Compaction energy	3		5	
	Water content *w* (%)	Dry density *ρ*_*d*_ (g/cm^3^)	Water content *W* (%)	Dry density *ρ*_*d*_ (g/cm^3^)
1208.2	10.07	1.746	10.05	1.717
	12.06	1.780	11.54	1.775
	13.50	1.795	13.81	1.815
	15.91	1.755	15.86	1.757
2013.7	9.50	1.758	10.03	1.783
	11.19	1.806	12.87	1.848
	12.54	1.808	14.13	1.823
	15.47	1.765	15.14	1.775
2684.9	9.82	1.788	9.83	1.795
	11.74	1.827	12.33	1.867
	13.82	1.809	13.90	1.840
	15.88	1.738	15.42	1.789

As shown in Fig 2(A) and 2(B), the dry densities of the soil under equal compaction energies and soil layers first increased with increasing water content and then decreased with increasing water content after reaching the optimal level. The abscissa corresponding to the peak density is the optimal water content ***w***_***op***_ (%) of the soil sample under the corresponding compaction energy, and the ordinate corresponds to the maximum dry density ***ρ***_***dmax***_ (g/cm^3^). With increasing compaction energy, the optimal water content of the soil decreases and the maximum dry density increases, in agreement with the compaction curve characteristics of fine-grained soils. The optimal water contents and maximum dry densities under different conditions are listed in Table 5.

**Table 5:** Optimum water content and maximum dry density under different compaction energies and soil layers.

Soil layers *n* *E* (kJ/m^3^) Compaction energy	3		5	
	Optimal water content *w_op_* (%)	Maximum dry density *ρ_dmax_* (g/cm^3^)	Optimal water content *w_op_* (%)	Maximum dry density *ρ_dmax_* (g/cm^3^)
1208.2	13.5	1.795	13.8	1.815
2013.7	12.5	1.810	12.8	1.850
2684.9	11.8	1.830	12.3	1.867

**Fig 3:**
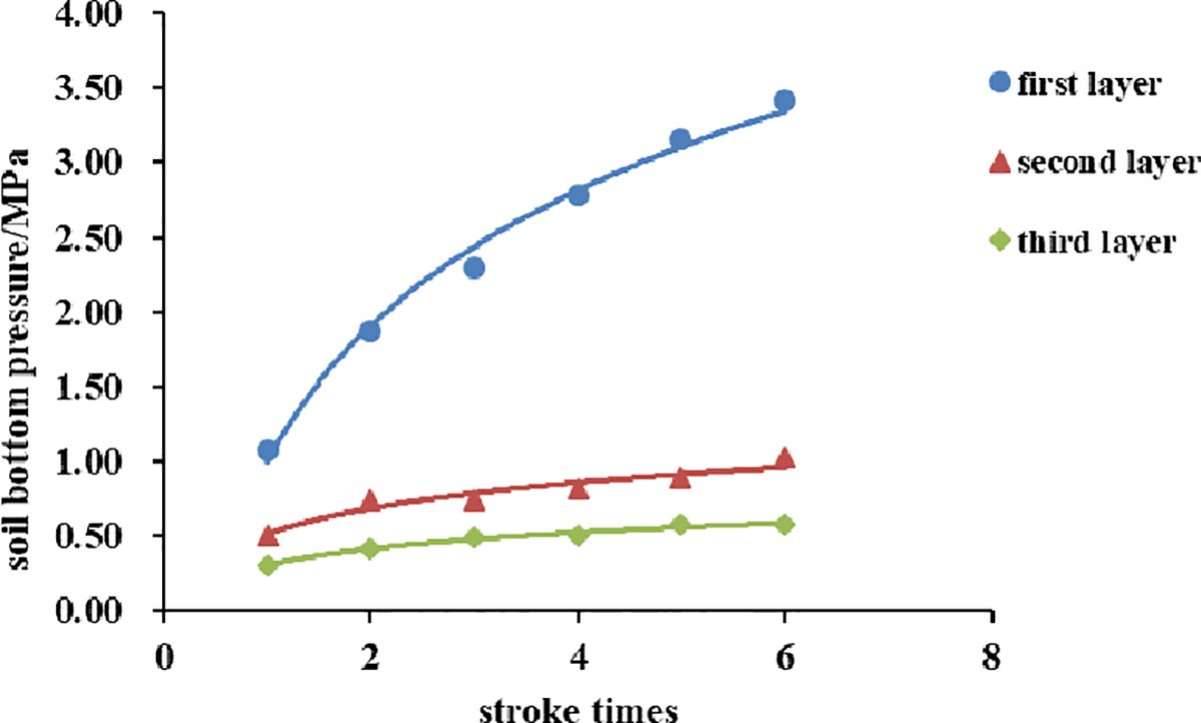
Variation curve of soil bottom pressure with the number of hammer strokes.

In the compaction tests of the six groups under different conditions, the soil bottom pressure of each layer is measured by the soil pressure box at the bottom of the soil. At the same time, the hammer impact force is measured by the impact force sensor during compaction. Because the compaction and remolding process of each layer of soil is similar [23], the changes in soil bottom pressure for each layer are also similar. Fig 3 shows the variation curve of the soil bottom pressure at the optimal water content with the number of hammer strokes of each soil sample (corresponding to the last stroke of each cycle), with a compaction energy of 2684.9 kJ/m^3^ and three soil layers.

Fig 3 shows that the soil bottom pressure is increased with an increasing number of hammer strokes, reaching the maximum at the last stroke of each layer. During testing, as the number of strokes is increased, the soil becomes increasingly compact, compaction energy is not used for compaction of the soil, but is transmitted directly to the bottom of the soil through a stable soil skeleton, and the impact force transferred to the soil bottom increases. As the number of soil layers is increased, the soil thickness increases, and the soil bottom pressure continues to increase relative to the number of strokes of one layer, but it increases more slowly with an increasing number of strokes, and the maximum value noticeably decreases. This analysis shows that the increase in soil thickness increases the path length of the impact force transferred to the bottom of the soil. Because the effective action depth of the force is fixed, the force transferred to the soil bottom decreases, so the soil bottom pressure also decreases.

**Fig 4:**
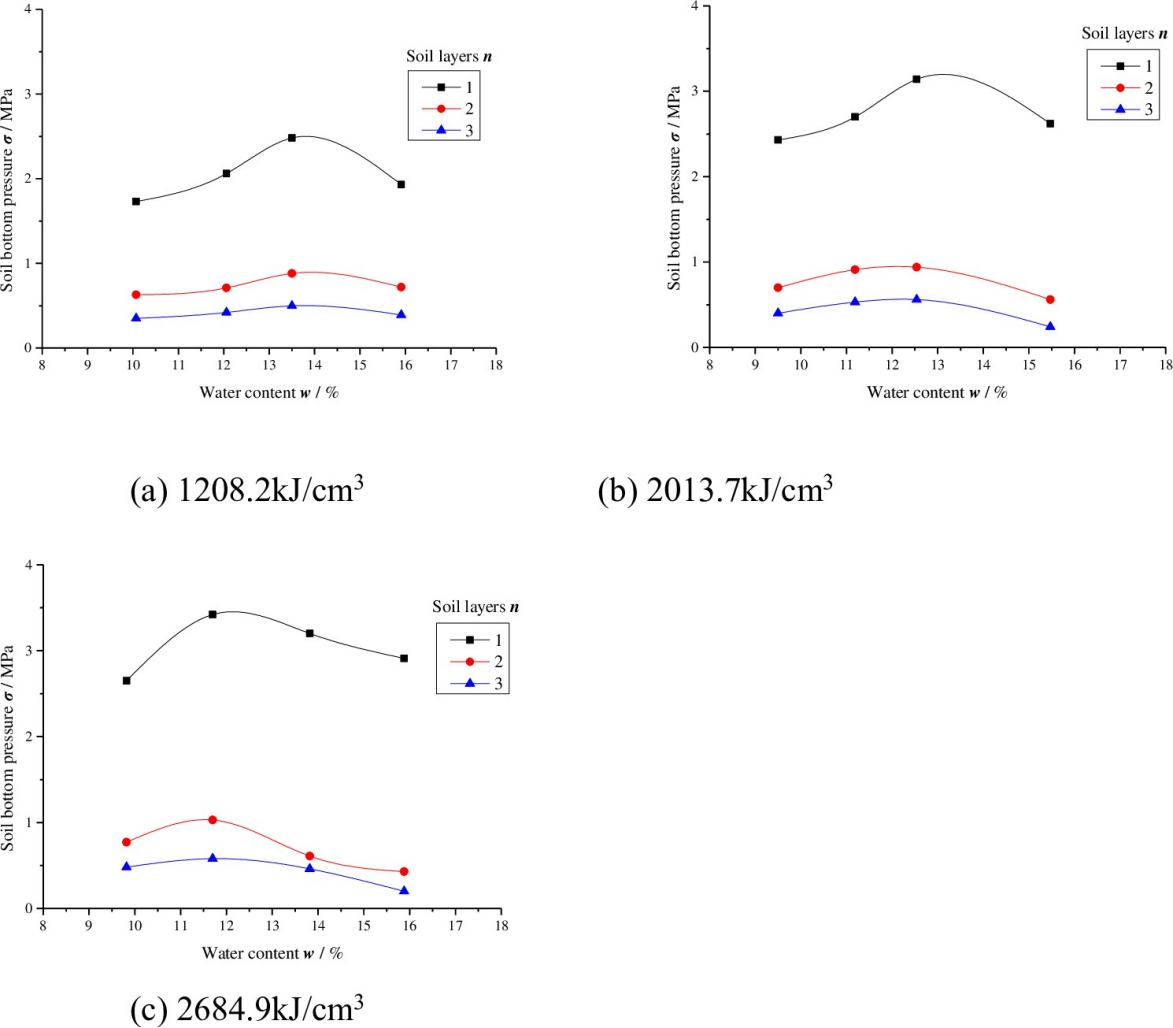
The soil bottom pressure of different compaction energies in the three-layer compacted soil sample (*E* = 1208.2 kJ/m^3^, 2013.7 kJ/m^3^, 2684.9 kJ/m^3^).

**Fig 5:**
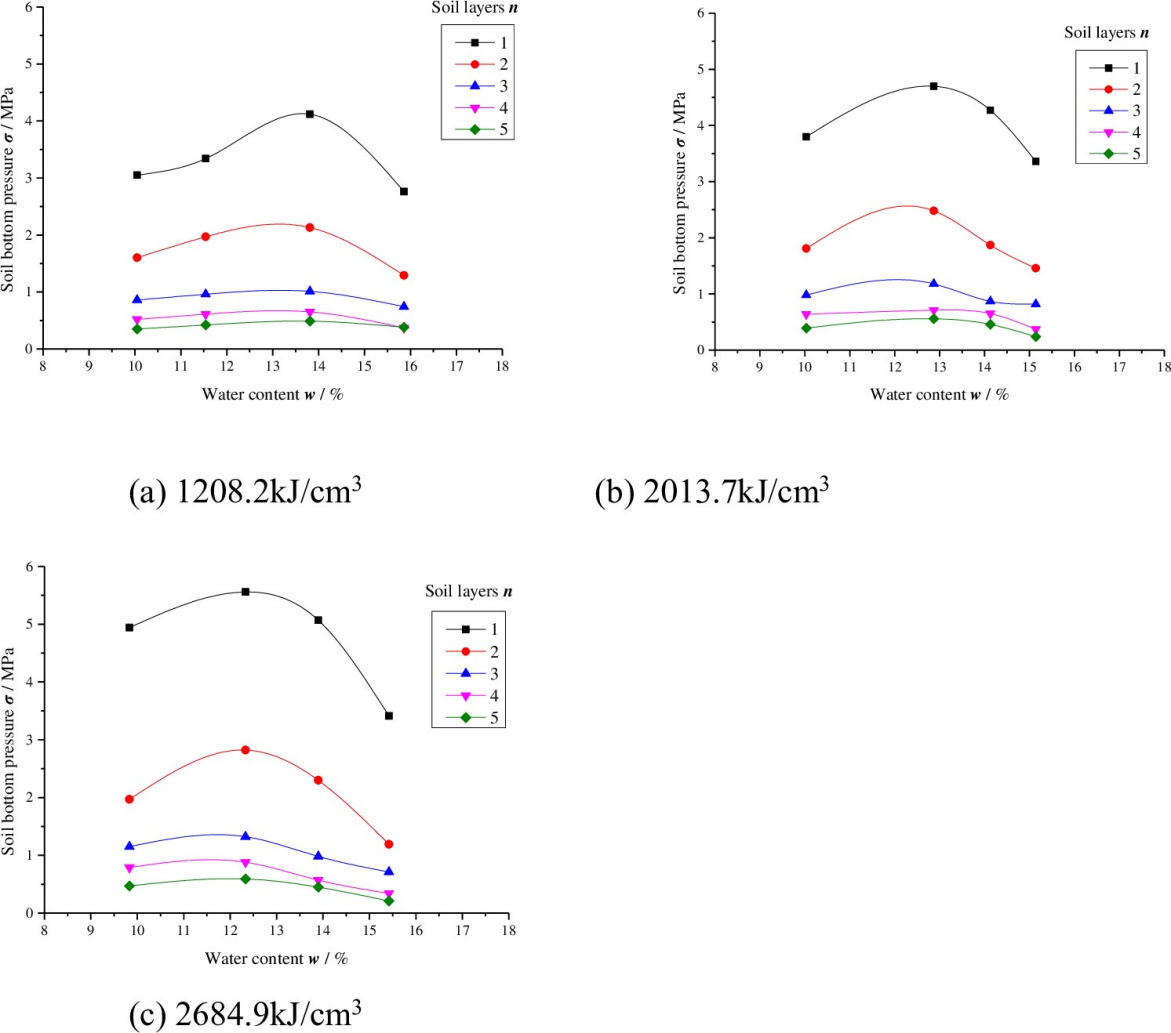
The soil bottom pressure of different compaction energies in the five-layer compacted soil sample (*E* = 1208.2 kJ/m^3^, 2013.7 kJ/m^3^, 2684.9 kJ/m^3^).

For the sake of research on the change in energy transfer in the process of compaction, the impact force of the hammer and the soil bottom pressure were recorded when the hammer stroke the soil, the maximum soil bottom pressure ***σ*** (MPa) of each layer of soil and the corresponding value of the hammer impact force ***σ***_***z***_ (MPa) (i.e., the maximum hammer impact force) were taken for analysis. The specific data are shown in Tables 6 and 7. The relationships between the soil bottom pressure values and water content are shown in Figs 4(A)–4(C) and 5(A)–5(C).

**Table 6:** The *σ*, *σ*_*z*_ and *σ/σ*_*z*_ values of each layer of soil in the three-layer soil sample.

Compaction energy *E* (kJ/m^3^)	Water content *w* (%)	Soil layers *n*								
		1			2			3		
		soil bottom pressure *σ*	Hammer impact force *σ*_*z*_	Energy consumption ratio *σ/σ*_*z*_	soil bottom pressure *σ*	Hammer impact force *σ*_*z*_	Energy consumption ratio *σ/σ*_*z*_	soil bottom pressure *σ*	Hammer impact force *σ*_*z*_	Energy consumption ratio *σ/σ*_*z*_
1208.2	10.07	1.73	6.09	0.28	0.63	3.16	0.20	0.35	0.35	0.16
	12.06	2.06	6.13	0.34	0.71	6.10	0.12	0.42	0.42	0.07
	13.50	2.48	6.22	0.40	0.88	6.17	0.14	0.50	0.50	0.08
	15.91	1.93	6.14	0.31	0.72	6.09	0.12	0.39	0.39	0.06
2013.7	9.50	2.43	6.11	0.40	0.70	4.18	0.17	0.40	0.40	0.13
	11.19	2.70	6.12	0.44	0.91	6.12	0.15	0.53	0.53	0.09
	12.54	3.14	6.15	0.51	0.94	6.31	0.15	0.56	0.56	0.09
	15.47	2.62	6.10	0.43	0.56	2.73	0.21	0.24	0.24	0.14
2684.9	9.82	2.65	6.17	0.43	0.77	6.11	0.13	0.48	0.48	0.08
	11.70	3.42	6.19	0.55	1.03	6.13	0.17	0.58	0.58	0.09
	13.82	3.20	6.08	0.53	0.61	6.14	0.10	0.46	0.46	0.08
	15.88	2.91	6.13	0.48 s	0.43	3.30	0.13	0.20	0.20	0.10

**Table 7:** The *σ*, *σ*_*z*_ and *σ/σ*_*z*_ values of each layer of soil in the five-layer soil sample.

*E* (kJ/m^3^)	*W* (%)	*N*														
		1			2			3			4			5		
		*σ*	*σ*_*z*_	*σ/σ*_*z*_	*σ*	*σ*_*z*_	*σ/σ*_*z*_	*σ*	*σ*_*z*_	*σ/σ*_*z*_	*σ*	*σ*_*z*_	*σ/σ*_*z*_	*σ*	*σ*_*z*_	*σ/σ*_*z*_
1208.2	10.05	3.05	6.09	0.50	1.60	5.12	0.31	0.86	4.14	0.21	0.52	3.19	0.16	0.35	2.73	0.13
	11.54	3.34	6.09	0.55	1.97	6.11	0.32	0.96	6.09	0.16	0.61	6.15	0.10	0.42	6.14	0.07
	13.81	4.12	6.17	0.67	2.13	6.18	0.35	1.01	6.12	0.17	0.65	6.09	0.11	0.49	6.11	0.08
	15.86	2.76	5.92	0.47	1.29	6.14	0.21	0.74	6.14	0.12	0.37	6.10	0.06	0.38	6.15	0.06
2013.7	10.03	3.80	6.12	0.62	1.81	6.13	0.29	0.98	6.08	0.16	0.64	6.14	0.10	0.39	6.08	0.06
	12.87	4.70	6.18	0.76	2.48	6.15	0.40	1.18	6.08	0.19	0.71	6.09	0.12	0.56	6.12	0.09
	14.13	4.27	6.14	0.70	1.87	6.09	0.31	0.87	6.14	0.14	0.65	6.15	0.11	0.46	6.20	0.07
	15.14	3.36	6.11	0.55	1.46	4.19	0.35	0.82	3.66	0.22	0.37	3.19	0.12	0.24	2.74	0.09
2684.9	9.83	4.94	6.12	0.81	1.97	6.11	0.32	1.15	6.13	0.19	0.79	6.11	0.13	0.47	6.12	0.08
	12.33	5.56	6.20	0.90	2.82	6.14	0.46	1.32	6.12	0.22	0.88	6.11	0.14	0.59	6.08	0.10
	13.9	5.07	6.13	0.83	2.30	6.12	0.38	0.98	6.11	0.16	0.57	6.12	0.09	0.45	6.09	0.07
	15.42	3.41	6.09	0.56	1.19	4.63	0.26	0.71	3.66	0.19	0.34	2.48	0.14	0.21	1.98	0.11

Figs 4 and 5 show that under equal compaction energy, in each layer of soil, ***σ*** first increases with increasing water content and then decreases with increasing water content above the optimal level and maximum pressure. The peak value in each layer of soil occurs at the optimal water content and thus the highest soil dry density. This is because under the same compaction energy, when the water content in each layer of soil is low, there is a large friction resistance between particles, which is not easily compacted. At the beginning of the compaction, the soil is in a loose state. According to Biot’s consolidation theory, the contact surface of soil particles per unit area is small, so the force transmitted to the bottom of the soil is small, and the pressure value at the bottom of the soil is low. Most of the impact force is absorbed by the soil particles to overcome the resistance between particles. As the water content increases, water on the particle surfaces begins to play a lubricating role, reducing the resistance between grains. Under the action of compaction, the loose soil particles gradually form a stable soil skeleton and the contact area of soil particles increases, so the energy transferred to the bottom of the soil is increasing. At water contents exceeding the optimum level (> ***w***_***op***_), the air in the soil is sealed and cannot dissipate during compaction. When the impact force is applied to the soil, most of the force is borne by pore water and pore gas, but only a small part is transferred to the bottom of the soil, and the pressure at the bottom of the soil decreases. The variation in the maximum soil bottom pressure ***σ*** of each layer with water content also reveals the compaction properties of fine-grained soil from the perspective of energy transfer.

As seen from Tables 6 and 7, the maximum impact force value of each layer of soil remained basically unchanged near 6.10 MPa at approximately the optimal water content (***w***_***op***_ ± 2%). However, when the water content was too low or too high, the impact force decreased significantly. This is because the soil is easily compacted near the optimal water content. When the soil reaches a relatively dense state, the force reflected from the soil surface to the hammer bottom remains unchanged. However, when the water content is too low or too high, compaction is hindered. The soil is relatively loose, and the reaction force on the compaction hammer is small, so the impact force is low.

To study the energy transfer efficiency during compaction, soil samples with different compaction energies under the condition of optimal water content were analyzed. When the soil reaches the maximum dry density ***ρ***_***dmax***_ under the corresponding conditions, the mechanical parameters of the soil are relatively stable. When the impact force is applied to the soil, the stress in the soil does not laterally diffuse but only transmits downward, and the stress diffusion angle can be ignored [24]. Therefore, the ratio of the maximum soil bottom pressure ***σ*** of each layer to the corresponding value of the hammer impact force ***σ***_***z***_ is defined as the soil bottom energy consumption ratio (***σ/σ***_***z***_). The soil bottom energy consumption ratios under different compaction energies (***w*** = ***w***_***op***_) are shown in Tables 6 and 7.

**Fig 6:**
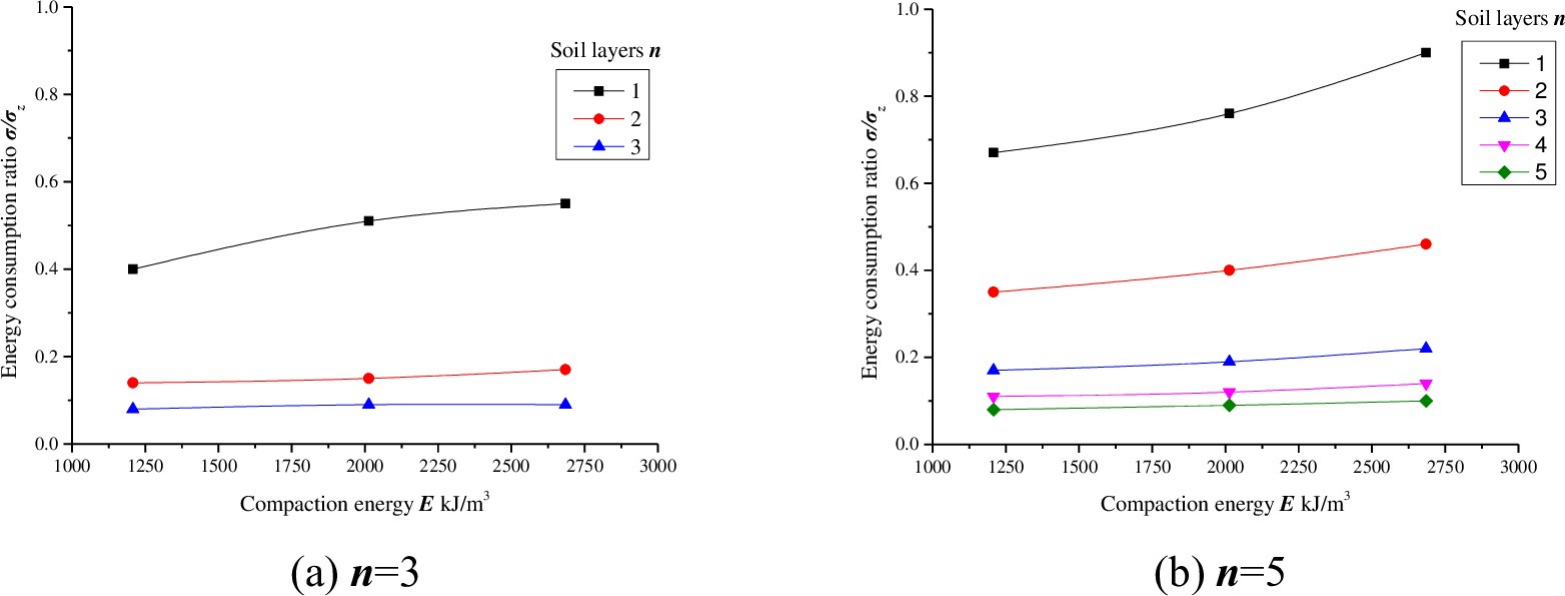
The soil bottom energy consumption ratio (*σ/σ*_*z*_) under different compaction energies (*E* = 1208.2 kJ/m^3^, 2013.7 kJ/m^3^, 2684.9 kJ/m^3^, *w* = *w*_*op*_).

Fig 6 shows that regardless of the soil layer, the value of ***σ/σ***_***z***_ in each layer increases with the compaction energy, but the trend is not significant. Since the impact force ***σ***_***z***_ per hammer stroke is constant, the higher the value of ***σ/σ***_***z***_ is, the greater the value of ***σ***. The process of increasing the compaction energy in the laboratory compaction test is obtained by the cumulative number of strokes. When the compaction energy increases, the number of strokes per layer increases, the particles are more closely packed together, the dry density is greater, and therefore more energy is transferred to the soil bottom at the same thickness. However, as the compaction energy continues to increase, the change in the ***σ/σ***_***z***_ value slows, meaning that increasing the compaction energy does not allow greater energy absorption into the soil, resulting in a waste of energy.

**Fig 7:**
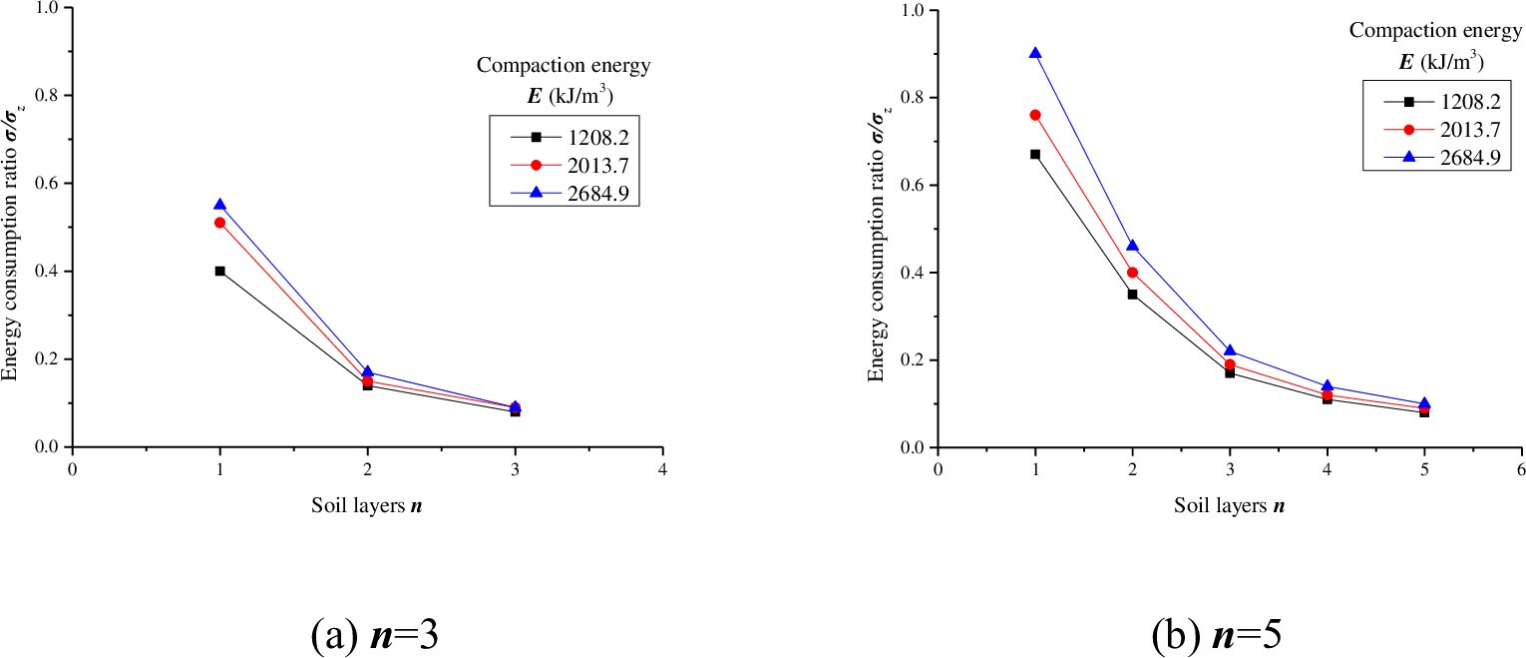
The soil bottom energy consumption ratio (*σ/σ*_*z*_) under different soil layers.

As seen from Figs 3–7, with the same compaction energy, the ***σ/σ***_***z***_ value of each layer decreases as the number of soil layers increases, but the decrease decreases and finally approaches zero. The changes under different conditions were fitted, and the results are shown in Table 8.

**Table 8:** The fitting results of each condition.

Compaction energy *E* (kJ/m^3^)	Soil layers *n*			
	3		5	
	Fitting equation	*R*^*2*^	Fitting equation	*R*^*2*^
1208.2	***σ/σ***_***z***_ = 0.824e^-0.805n^	0.97	***σ/σ***_***z***_ = 1.032e^-0.541n^	0.98
2013.7	***σ/σ***_***z***_ = 1.078e^-0.867n^	0.95	***σ/σ***_***z***_ = 1.179e^-0.547n^	0.97
2684.9	***σ/σ***_***z***_ = 1.243e^-0.905n^	0.97	***σ/σ***_***z***_ = 1.408e^-0.558n^	0.98

The relationship between the soil bottom energy consumption ratio (***σ/σ***_***z***_) and the soil layers can be summarized as follows:

$$\sigma /\sigma_{z}=k\prime e^{kn}$$ (3)

Under the condition of equal compaction energy, with the increase in the soil layers, the soil bottom energy consumption ratio decreases, and the change is similar to an exponential function. When ***n*** = 3, the range of variation in the ***n***-related coefficient ***k*** is larger, and the range is more than 10%. When ***n*** = 5, ***k*** is basically stable. The experiment reported here shows that the soil formed by five layers of compaction is more uniform and the soil skeleton is more stable than that formed by three layers of compaction. The coefficient ***k'*** is the correlation coefficient of compaction energy ***E***. It can be seen that, whether ***n*** = 3 or 5, ***k'*** increases linearly with the increase in ***E***, and the range of increase is the same. Therefore, under the experimental conditions in this paper, the compaction effect obtained by five layers is better than that obtained by three layers.

Based on the above conclusions, the comprehensive effects of compaction energy, water content, and soil layer number should be considered when soil compaction is performed on site. As the compaction energy increases, the dry density of the soil increases, but the growth rate gradually decreases, and the strength of the soil decreases with increasing dry density after reaching a certain limit, thus yielding excessive compaction. The compaction effect is better near the optimal water content. Insufficient water content corresponds to a large resistance between grains and difficult soil compaction. Excessive water corresponds to incomplete compaction and soft elastic phenomena. During compaction, the soil layer thickness should be monitored. Excessive thickness blocks the transfer of impact force to the bottom of the soil layer, yielding a loose structure at the bottom, and the soil skeleton is unstable. Excessive thinness and compaction energy lead to a waste of compaction energy. Therefore, the compaction energy, water content, and soil layer thickness must be considered comprehensively for compaction processes.

## Conclusion

This paper studies the relationship between compaction impact force and soil bottom pressure under different compaction energies and soil layers and determines the energy transfer process under various compaction conditions. Under the condition in this paper that both the bottom and the side of the soil sample are rigidly constrained, the following useful conclusions were obtained through research and analysis, and reasonable suggestions are proposed for engineering practice:

- During compaction, the soil bottom pressure in each layer increases with the number of hammer strokes. When the soil reaches the maximum dry density, the soil bottom pressure is the maximum.
- In each layer of soil under equal compaction energy, the variation curve of the soil bottom pressure with water content is similar to the compaction curve. This can reveal the compaction properties from the perspective of energy transfer.
- Under the optimal water content conditions of different compaction energies, the soil bottom energy consumption ratio in each layer increases slightly with increasing compaction energy and finally tends to remain unchanged. However, as the compaction energy of each stroke is the same, blindly increasing the compaction energy will lead to energy waste.
- Under the condition of equal compaction energy, with the increase in the number of the soil layers, the soil bottom energy consumption ratio decreases, and the change is similar to an exponential function. When the energy consumption ratio approaches 0, the compaction energy has reached the maximum depth of action. Under the experimental conditions, the soil skeleton is more stable after five layers of compaction than after three layers of compaction.

## Supporting information

S1 File Certificate of soil materials. (PDF)
