# Supplementary material for: Energy transfer and influencing factors in soil during compaction
Source: PLoS One. 2020 Nov 20;15(11):e0242622. doi: 10.1371/journal.pone.0242622 (PMC7678969; doi:10.1371/journal.pone.0242622)
Supplement: S1 File — (PDF) [file pone.0242622.s001.pdf]

Certificate of soil materials

The purpose of this letter is to certify that the soil materials used in this paper (**PONE-D-20-07195R1**) is approved for collection. And the Shanxi Survey Design Research Institute CO., LTD is responsible for collecting soil materials. The soil field is located in Ningwu County, Xinzhou City, Shanxi Province, China, with the geographical coordinates of 111°50'-120°40' east longitude and 38°31'-39°8' north latitude.

Please don’t hesitate to contact us if you have any questions about the certificate.

Best Regards.

Name of the leader: Jin Liang

Position of the leader: Vice President of engineering

Signature:

Tel: 0351- 4434030

Add: 57 Dunhua south road, Taiyuan, Shanxi Province, China

Institute: Shanxi Survey Design Research Institute CO., LTD
